# Joint effects of visual acuity impairment and visual field loss on reading performance: a low-vision simulation in Chinese readers

**DOI:** 10.1186/s40662-026-00497-x

**Published:** 2026-06-12

**Authors:** Xuhong Luo, Sijie Ye, Bangyan Sun, Yuanyuan Liu, Xiaoyue Hu, Lizhong Wang, Xiaoman Li, Jie Chen

**Affiliations:** 1 National Clinical Research Center for Ocular Diseases, Wenzhou, 325027 Zhejiang China; 2 Eye Hospital and School of Ophthalmology and Optometry, Wenzhou Medical University, Wenzhou, 325027 Zhejiang China

**Keywords:** Visual impairment, Low vision, Visual acuity, Visual field, Reading performance, Reading speed

## Abstract

**Background:**

This study aimed to evaluate the individual and joint effects of reduced visual acuity (VA) and restricted visual field (VF) on the reading performance of Chinese readers under standardized low-vision simulation.

**Methods:**

Eighty-one adults were randomized to three simulated distance VA levels using Bangerter foils: normal VA (≤ 0.30 logMAR), mild VA impairment (VAI) (0.30 logMAR< VA ≤ 0.52 logMAR), and moderate VAI (> 0.52 logMAR). VF was simulated as normal, moderate VF impairment (VFI) (20° radius), or severe VFI (10° radius) using specially designed goggles. Reading speed (RS) was measured using the Chinese International Reading Speed Texts (IReST). Maximum reading speed (MRS), reading acuity (RA), and critical print size (CPS) were measured using the Chinese Reading Acuity Chart. RS and MRS were analyzed using linear mixed-effects models, whereas RA and CPS were analyzed using generalized estimating equations with a gamma distribution and log link.

**Results:**

Reduced VA and restricted VF significantly decreased RS and MRS. Compared with normal VA, mild VAI reduced RS by 80.6 characters per minute (cpm, 95% CI: − 98.4 to − 62.7) and MRS by 52.9 cpm (95% CI: − 65.3 to − 40.6), while moderate VAI reduced MRS by 63.3 cpm (95% CI: − 75.6 to − 50.9). Moderate VFI and severe VFI reduced RS by 9.5 cpm (95% CI: − 16.7 to − 2.4) and 38.1 cpm (95% CI: − 45.3 to − 31.0), and reduced MRS by 11.0 cpm (95% CI: − 19.0 to − 3.1) and 25.2 cpm (95% CI: − 33.1 to − 17.3), respectively. Under combined impairment, participants with mild VAI and severe VFI demonstrated an additional reduction in RS of − 20.1 cpm (95% CI: − 30.2 to − 10.0). Additional reductions in MRS were observed in participants with moderate VAI combined with moderate VFI (− 20.6 cpm; 95% CI: − 31.8 to − 9.4) and severe VFI (− 40.0 cpm; 95% CI: − 51.2 to − 28.8). RA and CPS were primarily influenced by VA. VF restriction only showed small effects on RA and no meaningful effect on CPS.

**Conclusions:**

Both reduced VA and VF constriction significantly impaired reading performance, with VA loss emerging as the dominant factor. Under standardized simulation of low-vision conditions, combined impairments produced additional reductions in RS and MRS. These findings support the evaluation of reading performance in low-vision rehabilitation and earlier visual rehabilitation in patients with combined VA and VF impairments.

**Trial registration:**

The study was registered with the Chinese Clinical Trial Registry (ChiCTR2400080206).

**Supplementary Information:**

The online version contains supplementary material available at 10.1186/s40662-026-00497-x.

## Background

Low vision, defined as reduced best-corrected visual acuity (VA) and/or restricted visual field (VF) that cannot be fully corrected with conventional medical or surgical treatment, can substantially impair quality of life [1, 2] and social participation [3]. Impaired reading ability is one of the most prevalent and impactful consequences of low vision, as reading is closely associated with education, employment, communication, and independent daily living [3, 4]. Previous studies have demonstrated that central vision impairment significantly reduces reading speed (RS) [5, 6] and worsens reading acuity (RA) and critical print size (CPS) [7]. Furthermore, peripheral VF constriction can profoundly disrupt normal eye movement patterns required for efficient reading [8, 9]. Patients with restricted VF often exhibit reduced overall reading efficiency and require more frequent eye and head movements.

Clinical practice suggests that a substantial proportion of patients with low vision experience concurrent reductions in VA and VF. For example, patients with glaucoma or retinitis pigmentosa frequently present with concomitant VA loss and VF restriction [8, 9]. However, most existing studies have assessed the effects of VA or VF impairment on reading performance separately, without accounting for their potential combined effects. Consequently, whether and how these deficits interact to influence reading impairment remains unclear. Quantifying the joint contributions of VA and VF deficits could provide important guidance for low-vision rehabilitation and inform how interventions targeting VA and VF should be balanced to optimize reading outcomes [5, 8, 9].

Significant interindividual heterogeneity among patients with low vision complicates rehabilitation strategies. Variability in underlying ocular diseases, the type and severity of visual impairment, and individual differences in how patients utilize residual vision all influence rehabilitation approaches [4]. This heterogeneity highlights the need for a standardized low-vision simulation model to evaluate reading performance while minimizing confounding factors. In the present study, two validated methods were used to simulate low-vision conditions: Bangerter diffusing foils to reduce VA and goggles with restricted apertures to simulate peripheral VF constriction. This simulation approach isolates the effects of VA and VF impairment in normally sighted adults and avoids confounding from individual visual impairment experience and adaptive recovery processes [10, 11].

Of note, most published studies have focused on alphabetic languages, particularly English [12]. By contrast, Chinese is a logographic writing system characterized by visually complex characters, the absence of interword spacing, and a unique mapping between orthography and phonology. These features result in a smaller perceptual span and distinct eye movement strategies compared with alphabetic reading [13]. Thus, visual impairments may affect reading performance differently in Chinese readers than in readers of alphabetic languages.

This study aimed to establish a standardized simulation model to investigate the joint effects of reduced VA and restricted VF on reading performance, thereby providing a reference for reading rehabilitation.

## Methods

### Study design

This was a randomized controlled simulation study. The participants were randomly assigned to one of three simulated distance VA levels, and each participant was tested under three simulated VF conditions. The randomization sequence was generated using a computer-based random number generator with a 1:1:1 allocation ratio. The study was approved by the Ethics Committee of the Eye Hospital of Wenzhou Medical University (Approval No. 2023-168-K-138). The trial was registered with the Chinese Clinical Trial Registry (Registration No. ChiCTR2400080206). Eligible participants met the following inclusion criteria: (1) binocular best-corrected distance VA of ≤ 0.10 logMAR and normal VF, (2) age between 18 and 40 years old, (3) native Chinese speaker, and (4) ability to understand study instructions and complete all test procedures. The exclusion criteria were as follows: (1) any ocular diseases, including cataract, glaucoma, corneal dystrophy, and nystagmus; (2) history of ocular surgeries; and (3) any systemic diseases affecting visual function. All procedures adhered to the tenets of the Declaration of Helsinki, and written informed consent was obtained from all participants.

### Visual function assessments and low vision simulation based on World Health Organization (WHO) categorization

The binocular initial VA (IVA) was measured using the Chinese national standard logarithmic distance VA chart (GB 11533–2011). Under each simulated condition, binocular-simulated distance VA (sDVA) was assessed using the same chart. Binocular simulated near VA (sNVA) was measured at 40 cm using the Chinese standard logarithmic near VA chart (GB 11533–2011). All VA measurements were expressed in logMAR units. Contrast sensitivity under simulated conditions was measured binocularly using the Mars Contrast Sensitivity Test (Mars Perceptrix, USA) and recorded in logarithmic units (logCS).

According to the WHO visual impairment classification, simulated VA levels were defined based on sDVA and categorized as normal VA (sDVA ≤ 0.30 logMAR), mild visual acuity impairment (VAI) (0.30 logMAR < sDVA ≤ 0.52 logMAR), and moderate VAI (sDVA > 0.52 logMAR). VF conditions were categorized as normal VF, moderate visual field impairment (VFI) (20° VF radius), and severe VFI (10° VF radius) [14]. The three VA levels and three VF levels were combined and mapped to five WHO visual impairment categories: normal vision, mild visual impairment, moderate visual impairment, severe visual impairment, and blindness (Supplementary Table 1). Bangerter occlusion foils were used to reduce the participants’ VA to the sDVA range. Foil density was selected and adjusted as needed, based on each participant’s sDVA to ensure classification within the predefined target category.

**Fig. 1:**
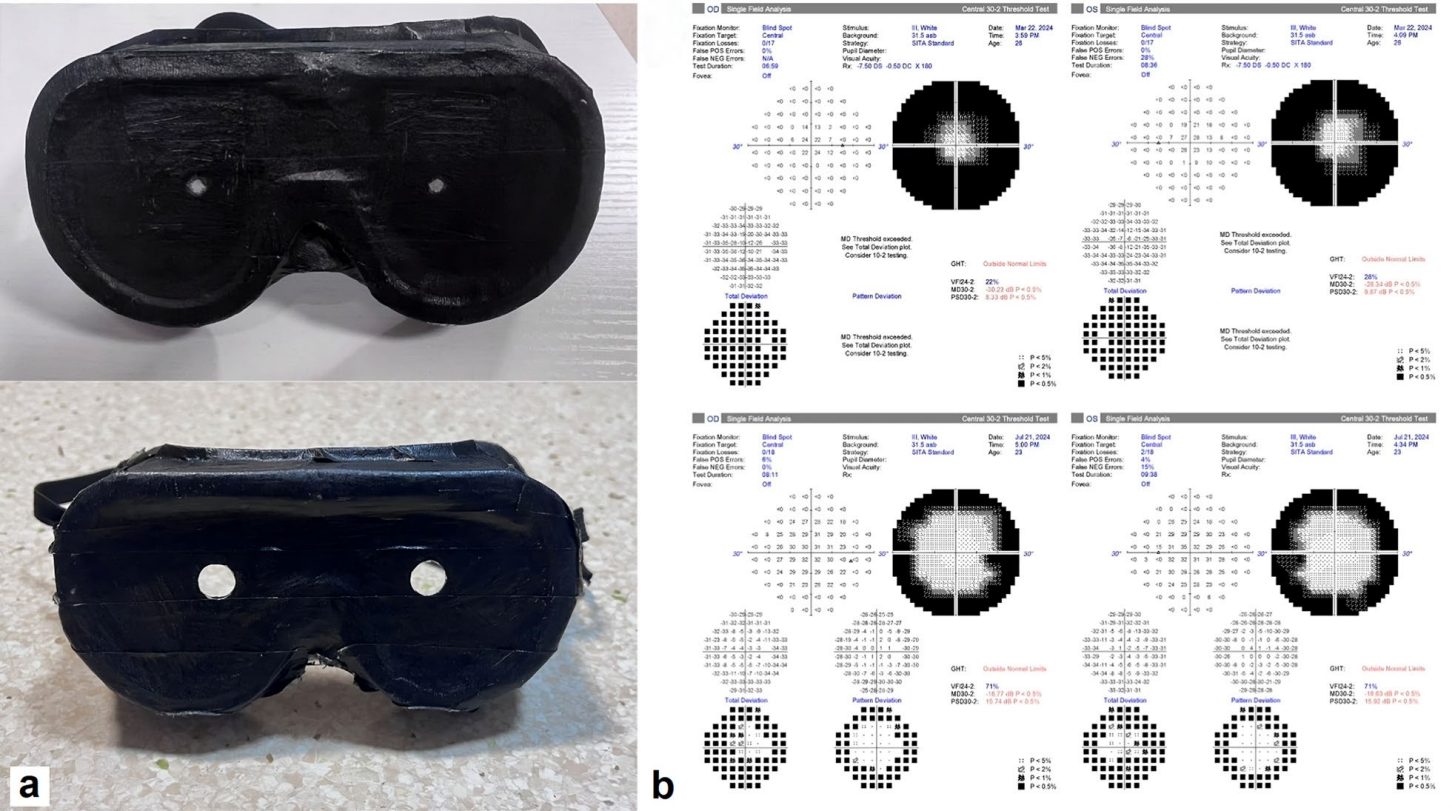
Validation of the visual field (VF) simulation system. **a** Schematic illustration of the VF-restriction goggles with circular apertures. Aperture radii were 5.2 mm (20° VF radius) and 2.6 mm (10° VF radius). **b** Humphrey VF testing confirmed the effectiveness of the VF restriction simulation. The VF patterns demonstrate successful simulation of 20° and 10° VF constriction

To simulate peripheral VF constriction, specially designed goggles with restricted circular apertures were used (Fig. 1a), generating three VF conditions. The aperture radius (r) on the goggle was obtained following the formula r = d*tan(θ), where θ represents the target VF radius (10° or 20°). The distance between the goggle aperture plane and the corneal apex was set at 12 mm. The entrance pupil was assumed to be located 3 mm posterior to the corneal apex. Accordingly, the distance from the goggle aperture plane to the entrance pupil center (d) was 15 mm. Based on this configuration, the corresponding aperture radii were 5.2 mm (20° VF radius) and 2.6 mm (10° VF radius). The effectiveness of the simulated VF restrictions was verified in each participant using a Humphrey VF analyzer (Fig. 1b).

The participants were randomly assigned to three VA simulation groups, and each participant completed the reading task under all three VF conditions (normal, 20°, and 10°).

## Reading ability assessment

### Reading speed

RS reflects the functional continuous-text reading performance at a fixed print size under standardized near-reading conditions. RS was assessed using the Chinese version of the International Reading Speed Texts (IReST), a standardized set of reading materials designed to evaluate RS [15]. The Chinese IReST consists of 10 short texts. Each text was presented on a single page, and all pages were randomly arranged to allow repeated measurements [16]. Each text contained 153 Chinese characters, and all texts were comparable in terms of difficulty and linguistic complexity. The reading distance was fixed at 40 cm for all RS assessments. As the IReST text could not be reliably read at 40 cm under the moderate VAI level across all VF conditions, this condition was excluded from RS analyses. The participants were instructed to maintain the specified reading distance and read aloud as quickly as possible without interruption from the examiner. Reading time was recorded, and misread or omitted characters were documented. The entire process was audio-recorded for subsequent verification. RS was calculated as follows:

RS (cpm) = (correct characters/reading time) × 60.

Correct characters = total characters − misread and omitted characters.

### Maximum reading speed (MRS), reading acuity, and critical print size

The MRS, RA, and CPS were measured using the Chinese Reading Acuity Chart, which was developed with consideration of character frequency, stroke count, and syntactic complexity (Supplementary Fig. 1) [17]. The readability of the chart closely approximates that of daily reading materials and is designed to evaluate functional reading ability in real-life conditions. The chart comprised three equivalent sets, each containing 13 optotypes of progressively decreasing print size. Each optotype comprises a 30-character sentence (including three punctuation marks), with adjacent print sizes differing by 0.1 log units (factor of 1.2589). The participants were instructed to read the sentences aloud from the largest to the smallest print size according to the standard protocol of the Chinese Reading Acuity Chart. Reading time and errors were recorded. RA was defined as the smallest print size that could be correctly recognized and was expressed in logMAR units. CPS was defined as the smallest print size, in logMAR, at which the participant’s RS reached its maximum value. The MRS was defined as the highest RS attained across all print sizes, reflecting peak reading performance when print size is not a limiting factor.

All visual function and reading tests were completed in a single session for each participant, with a total duration of approximately 30–40 min, including a 2–3-min break between conditions.

### Statistical analysis

All analyses were conducted using R (version 4.3.0; R Foundation for Statistical Computing, Vienna, Austria). The primary objective was to quantify the effects of simulated VA, VF, and their interaction on reading outcomes, while accounting for repeated measures within participants. RS and MRS were analyzed using linear mixed-effects models with a participant-level random intercept. RA and CPS were analyzed using generalized estimating equations (gamma distribution with log link) to obtain population-averaged estimates while accounting for within-subject correlations. Bonferroni correction was applied to all fixed-effect *P* values, with significance defined as *P* < 0.05. Model-based marginal means and post hoc contrasts are presented in the corresponding tables and figures.

## Results

Eighty-one participants completed the study, with 27 randomly assigned to each of the three VA groups. No significant differences were observed among the three groups in terms of sex distribution, age, or baseline VA prior to simulation (all *P* > 0.05). The median IVA (logMAR) before simulation was − 0.08 (interquartile range [IQR]: − 0.08, 0.00) across all groups. After application of Bangerter foils, the median sDVA values (logMAR) were − 0.08 (IQR: − 0.08, 0.00), 0.40 (IQR: 0.40, 0.52), and 0.70 (IQR: 0.70, 0.82) in the normal VA, mild VAI, and moderate VAI groups, respectively. The corresponding median sNVA values (logMAR) were 0.00 (IQR: − 0.08, 0.00), 0.40 (IQR: 0.40, 0.52), and 0.70 (IQR: 0.70, 0.82), respectively (Table 1).

**Table 1:** Baseline demographic and visual characteristics

Parameters	Groups			χ^2^/F/H	*P*
	Normal VA (n = 27)	Mild VAI (n = 27)	Moderate VAI (n = 27)		
Male [n (%)]	13 (48.1)	12 (44.4)	12 (44.4)	0.100^a^	0.951
Female [n (%)]	14 (51.9)	15 (55.6)	15 (55.6)	-	-
Age (mean ± SD)	26.37 ± 2.45	26.41 ± 2.96	26.33 ± 3.03	0.005^b^	0.995
IVA [median (Q1, Q3)]	− 0.08 (− 0.08, 0.00)	− 0.08 (− 0.08, 0.00)	− 0.08 (− 0.08, 0.00)	0.697^c^	0.706
sDVA [median (Q1, Q3)]	− 0.08 (− 0.08, 0.00)	0.40 (0.40, 0.52)	0.70 (0.70, 0.82)	73.182^c^	< 0.001
sNVA [median (Q1, Q3)]	0.00 (− 0.08, 0.00)	0.40 (0.40, 0.52)	0.70 (0.70, 0.82)	73.123^c^	< 0.001
LogCS [median (Q1, Q3)]	1.80 (1.80, 1.80)	1.54 (1.48, 1.60)	1.20 (0.98, 1.20)	70.857^c^	< 0.001

*IVA *= initial visual acuity (binocular distance VA before simulation); *sDVA* = simulated distance visual acuity; *sNVA* = simulated near visual acuity; *CS *= contrast sensitivity; VAI = visual acuity impairment. IVA, sDVA, and sNVA are expressed as logMAR units, whereas CS is expressed as logarithmic contrast sensitivity (logCS)

^a^Chi-square test; ^b^One-way ANOVA; ^c^Kruskal-Wallis test. *P* values in bold indicate statistical significance

### Reading speed

**Fig. 2:**
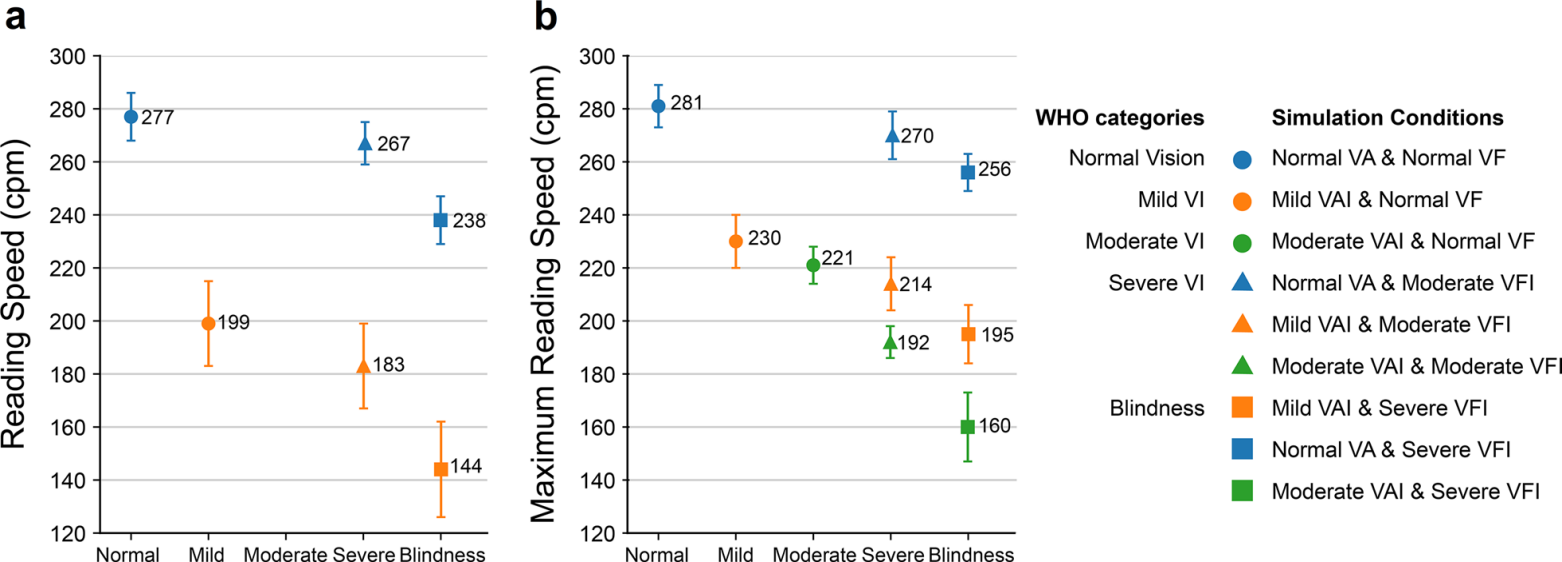
Reading performance under different simulated visual acuity (VA) and simulated visual field (VF) conditions. **a** Measured reading speed (RS) under different simulated conditions. **b** Measured maximum reading speed (MRS) under different simulated conditions. Data are expressed as means, and error bars indicate 95% confidence intervals. Blue symbols represent normal VA, orange symbols represent mild VA impairment (VAI), and green symbols represent moderate VAI. “●” indicates normal VF, “▲” indicates moderate VF impairment (VFI; 20° VF radius), and “■” indicates severe VFI (10° VF radius). The moderate VAI condition was not included in (**a**) because RS could not be measured at a viewing distance of 40 cm across all VF conditions. VI, visual impairment

The RS was approximately 277 characters per minute (cpm) in participants without visual impairment (i.e., normal VF and VA), which served as the reference group for subsequent comparisons. All measured values across visual function groups are presented in Fig. 2a and Supplementary Table 1. Compared with the reference group, participants in the mild VAI group read significantly slower by 80.56 cpm (95% CI: − 98.44 to − 62.67; *P* < 0.001). VF restriction was also associated with reduced RS, with decreases of 9.52 cpm (95% CI: − 16.67 to − 2.37; *P* = 0.045) in the moderate VFI group and 38.11 cpm (95% CI: − 45.26 to − 30.96; *P* < 0.001) in the severe VFI group (Table 2).

**Table 2:** Linear mixed-effects model for reading speed (cpm) at 40 cm

Predictor	Estimate	95% CI	*P*
Mild VAI	− 80.56	(− 98.44, − 62.67)	< 0.001
Moderate VFI	− 9.52	(− 16.67, − 2.37)	0.045
Severe VFI	− 38.11	(− 45.26, − 30.96)	< 0.001
(Mild VAI) × Moderate VFI	− 8.67	(− 18.77, 1.44)	0.464
(Mild VAI) × Severe VFI	− 20.07	(− 30.18, − 9.97)	< 0.001

*CI* = confidence interval; *VAI* = visual acuity impairment; *VFI* = visual field impairment

Reference categories were normal VA and VF. A linear mixed-effects model with a participant-level random intercept was used to account for within-subject correlations across repeated measures, with parameters estimated using restricted maximum likelihood (REML). The 95% CIs are unadjusted, model-based estimates. *P* values were adjusted for multiple comparisons using the Bonferroni correction across non-intercept fixed effects. *P* values in bold indicate statistical significance

**Fig. 3:**
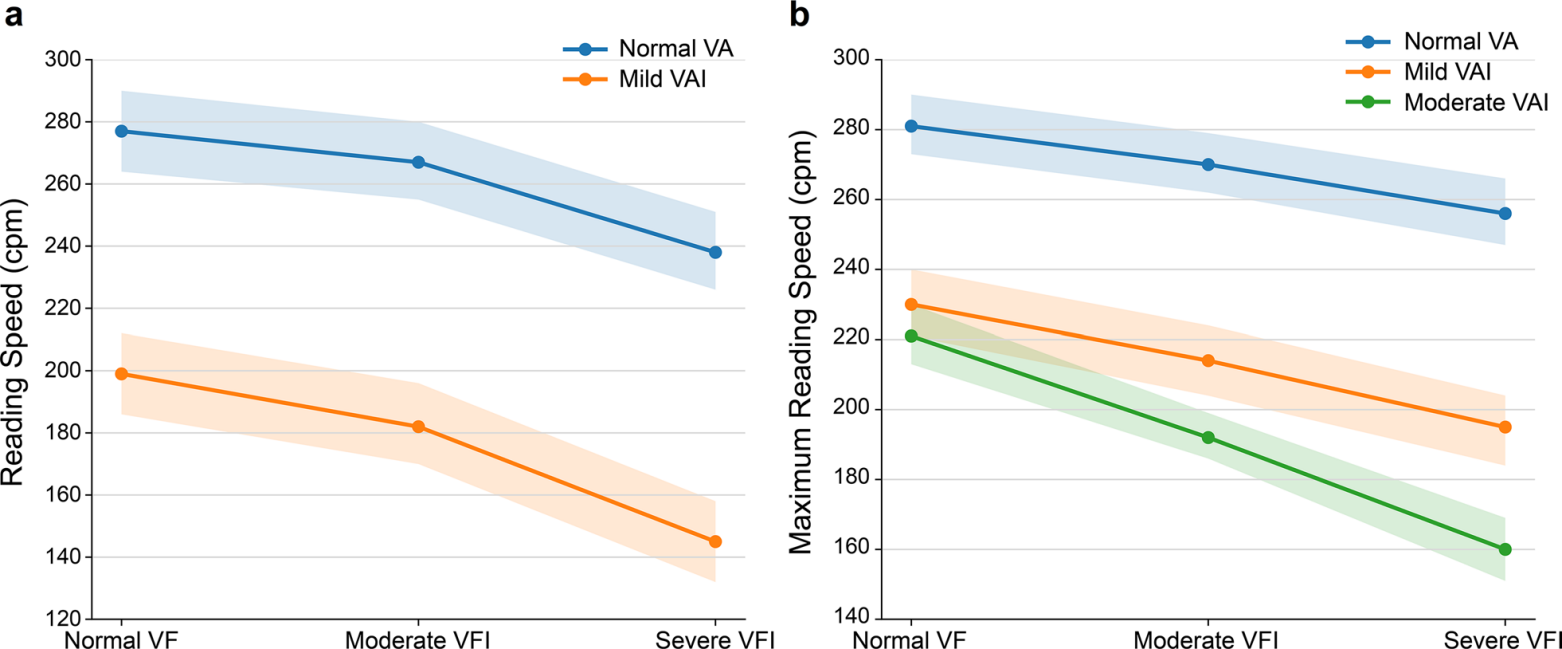
Interaction effects of simulated visual acuity (VA) and visual field (VF) conditions on reading performance. **a** Reading speed (RS). **b** Maximum reading speed (MRS). The x-axis represents VF conditions. Data points indicate model-based estimated marginal means for each VA condition, and shaded bands indicate 95% confidence intervals. The moderate VA impairment (VAI) condition is not shown in (**a**) because RS could not be measured at a viewing distance of 40 cm

A significant interaction effect was observed between mild VAI and severe VFI. Participants with both conditions had an RS that was 20.07 cpm lower (95% CI: − 30.18 to − 9.97; *P* < 0.001) than expected based on the sum of the individual main effects of mild VAI and severe VFI. No significant interaction effect was observed between mild VAI and moderate VFI (Table 2 and Fig. 3a).

### Maximum reading speed

The MRS was 281 cpm at a 40 cm reading distance in participants without visual impairment, which served as the reference for subsequent comparisons. All MRS values across visual function groups are shown in Fig. 2b and Supplementary Table 1. Compared with the reference group, participants in the mild VAI group showed a mean MRS reduction of 52.93 cpm (95% CI: − 65.26 to − 40.59; *P* < 0.001), whereas those in the moderate VAI group showed a mean MRS reduction of 63.26 cpm (95% CI: − 75.59 to − 50.93; *P* < 0.001). Compared with the reference group, participants with moderate VFI and severe VFI showed reductions in MRS of 11.04 cpm (95% CI: − 18.96 to − 3.12; *P* = 0.050) and 25.19 cpm (95% CI: − 33.10 to − 17.27; *P* < 0.001), respectively (Fig. 2b and Table 3).

**Table 3:** Linear mixed-effects model for maximum reading speed (cpm) at 40 cm

Predictor	Estimate	95% CI	*P*
Mild VAI	− 52.93	(− 65.26, − 40.59)	< 0.001
Moderate VAI	− 63.26	(− 75.59, − 50.93)	< 0.001
Moderate VFI	− 11.04	(− 18.96, − 3.12)	0.050
Severe VFI	− 25.19	(− 33.10, − 17.27)	< 0.001
Mild VAI × Moderate VFI	− 6.26	(− 17.46, 4.94)	> 0.99
Mild VAI × Severe VFI	− 12.15	(− 23.35, − 0.95)	0.268
Moderate VAI × Moderate VFI	− 20.59	(− 31.79, − 9.39)	0.003
Moderate VAI × Severe VFI	− 40.00	(− 51.20, − 28.80)	< 0.001

*CI *= confidence interval; *VAI *= visual acuity impairment; *VFI *= visual field impairment

Reference categories were normal VA and VF. A linear mixed-effects model with a participant-level random intercept was used to account for within-subject correlations across repeated measures, with parameters estimated using restricted maximum likelihood (REML). The 95% CIs are unadjusted, model-based estimates. *P* values were adjusted for multiple comparisons using the Bonferroni correction across non-intercept fixed effects. *P* values in bold indicate statistical significance

A combined effect was observed between moderate VAI and VF loss. Participants with both moderate VAI and moderate VFI showed an additional reduction in MRS of 20.59 cpm (95% CI: − 31.79 to − 9.39; *P* = 0.003) compared with the sum of individual effects. Similarly, participants with both moderate VAI and severe VFI showed an additional reduction of 40.00 cpm (95% CI: − 51.20 to − 28.80; *P* < 0.001) compared with either condition alone (Table 3 and Fig. 3b).

### Reading acuity and critical print size

Both RA and CPS were strongly dependent on VA. RA (logMAR) was approximately 2.3-fold higher in the mild VAI group (expβ = 2.319, *P* < 0.001) and approximately 3.3-fold higher in the moderate VAI group (expβ = 3.280, *P* < 0.001) compared with the normal VA group. CPS (logMAR) was approximately 1.8-fold higher in the mild VAI group (expβ = 1.831, *P* < 0.001) and approximately 2.1-fold higher in the moderate VAI group (expβ = 2.125, *P* < 0.001). VF restriction had no significant effect on CPS (Table 4).

**Table 4:** Generalized estimating equation models for reading acuity and critical print size

Predictor	RA			CPS		
	expβ	95% CI	*P*	expβ	95% CI	*P*
Mild VAI	2.319	(2.120, 2.537)	< 0.001	1.831	(1.536, 2.183)	< 0.001
Moderate VAI	3.280	(3.035, 3.545)	< 0.001	2.125	(1.796, 2.514)	< 0.001
Moderate VFI	1.062	(1.006, 1.120)	0.231	1.132	(0.887, 1.445)	> 0.99
Severe VFI	1.105	(1.033, 1.181)	0.028	1.191	(0.937, 1.514)	> 0.99
Mild VAI × Moderate VFI	0.954	(0.897, 1.014)	> 0.99	0.862	(0.669, 1.110)	> 0.99
Mild VAI × Severe VFI	0.968	(0.898, 1.044)	> 0.99	0.796	(0.623, 1.017)	0.545
Moderate VAI × Moderate VFI	0.928	(0.876, 0.983)	0.083	0.886	(0.694, 1.132)	> 0.99
Moderate VAI × Severe VFI	0.915	(0.848, 0.988)	0.180	0.848	(0.667, 1.079)	> 0.99

*CI* = confidence interval; *CPS* = critical print size; *RA* = reading acuity; *VAI *= visual acuity impairment; *VFI = *visual field impairment

Reference categories were normal VA and VF. As RA and CPS were repeatedly measured within participants, generalized estimating equations were used to obtain population-averaged estimates while accounting for within-subject correlations. A Gamma distribution with a log link and an exchangeable working correlation structure was specified. An exp (β) value of > 1 indicates multiplicative increases. The 95% CIs are unadjusted model-based intervals. *P* values were adjusted for multiple comparisons using the Bonferroni correction across non-intercept fixed effects. *P* values in bold indicate statistical significance

## Discussion

This study demonstrated that both reduced VA and restricted VF impaired reading performance among Chinese readers, with VA representing the dominant factor. The combined effects of VA and VF resulted in an additional reduction in reading performance beyond that observed with either impairment alone.

Consistent with prior findings in alphabetic languages, reduced VA decreased RS and MRS, while increasing both RA and CPS [6, 7, 15, 17]. The findings of the present study in Chinese readers further confirm the role of VA in reading performance. Adequate VA is a prerequisite for efficient reading, as image blur substantially impairs visual information processing in both alphabetic and logographic writing systems.

Compared with the pronounced impact of VA loss on reading, VF constriction resulted in only a modest reduction in reading performance. According to the WHO visual impairment classification, a VF radius of 10° falls within the category of blindness, regardless of VA level. However, in the present simulation, participants with severe VFI and normal VA read faster than those with moderate VAI and normal VF. These findings highlight the distinction between clinical definitions of visual impairment and task-specific functional vision. The WHO classification is primarily based on distance visual function and is intended to describe the severity of visual impairment; however, it does not necessarily predict near visual performance, such as continuous-text reading. Therefore, standardized reading assessments, such as continuous-text RS, are necessary [15, 18]. As compensatory eye and head movements cannot be fully eliminated in aperture-goggle simulations, reading performance under VF restriction may be overestimated. Nonetheless, this controlled paradigm provides useful insights for low-vision rehabilitation planning.

When RS and MRS were slightly reduced, threshold measures such as RA and CPS remained relatively stable, suggesting that participants with peripheral restriction maintained minimum print recognition by slowing their RS. In clinical cohorts with glaucoma and retinitis pigmentosa, restricted VF is associated with a reduced usable visual span and increased oculomotor demand, leading to slower text processing [8, 9, 19]. This may explain why VF restriction has a smaller effect on RS than VA impairment; however, this finding remains clinically important for assessing reading performance.

The most important finding of this study was the quantified synergistic effect of VA and VF impairments. Compared with either impairment alone, their combined effect resulted in additional reductions in reading performance, indicating a non-additive interaction. This phenomenon can be explained within the visual span framework. Visual blur reduces the ability to resolve fine detail, whereas VF loss restricts the number of visible characters and eliminates parafoveal preview information [20]. With fewer and less clearly resolved characters available, readers were forced to adopt a more serial reading strategy, which substantially limited RS [21]. This effect may be particularly relevant for Chinese reading. Chinese text consists of visually complex characters and is typically written without interword spacing [13, 17]. Reduced VA impairs character recognition, whereas VF restriction curtails parafoveal preview and the number of visible characters; together, these factors accelerate the shift toward serial reading and slow overall RS.

Based on the results of this study, interventions aimed at improving VA, such as magnification and contrast enhancement, should be prioritized in reading rehabilitation [22–24]. However, patients with combined VA and VF deficits require integrated rehabilitation strategies. For individuals with VF restriction, approaches that compensate for reduced visual span—such as training in head and eye movements and the use of scrolling text formats—should be considered [25, 26]. Early referral to vision rehabilitation may benefit patients with combined VA and VF impairments, such as those with glaucoma, by facilitating timely adoption of compensatory strategies and assistive devices, thereby improving daily functioning and quality of life [27].

Standardized reading assessments (IReST or MNREAD) should be incorporated alongside conventional letter-based VA measures in clinical evaluation, as standard visual function tests do not necessarily predict reading performance. Collectively, these findings provide a quantitative basis for balancing VA enhancement and VF compensation strategies when designing reading rehabilitation programs for Chinese readers with combined VA and VF impairments.

## Conclusions

VA loss, rather than VF restriction, was the primary factor impairing Chinese reading performance under standardized low-vision simulation conditions. In addition, combined VA loss and VF restriction produced interactive effects that further reduced both RS and MRS. These findings support the incorporation of standardized reading performance assessments into low-vision evaluation and rehabilitation planning, particularly for individuals with combined VA and VF impairments.

## Supplementary Information

Supplementary file1

## Data Availability

All data supporting the findings of this study are included in this article; additional datasets are available from the corresponding author on reasonable request.

## Abbreviations

VA: Visual acuity
VAI: Visual acuity impairment
VF: Visual field
VFI: Visual field impairment
RS: Reading speed
MRS: Maximum reading speed
RA: Reading acuity
CPS: Critical print size
IReST: International Reading Speed Texts
cpm: Characters per minute
