# Supplementary material for: Joint effects of visual acuity impairment and visual field loss on reading performance: a low-vision simulation in Chinese readers
Source: Eye Vis (Lond). 2026 Jun 12;13:25. doi: 10.1186/s40662-026-00497-x (PMC13262081; doi:10.1186/s40662-026-00497-x)

**S****upplementary Table 1** Mapping of simulated VA-VF conditions to WHO labels and corresponding estimated marginal means (EMMs) for RS and MRS

| **Simulated VA category** | **Simulated VF category** | **WHO label** | **RS EMM (cpm)** | **RS 95% CI** | **MRS EMM (cpm)** | **MRS 95% CI** |
| --- | --- | --- | --- | --- | --- | --- |
| Normal VA (VA [logMAR] ≤ 0.30) | Normal VF | Normal | 276.52 | (268.48, 284.56) | 281.41 | (274.02, 288.79) |
| Normal VA (VA [logMAR] ≤ 0.30) | Moderate VFI (20° radius) | Severe VI | 267.00 | (259.66, 274.34) | 270.37 | (262.28, 278.46) |
| Normal VA (VA [logMAR] ≤ 0.30) | Severe VFI (10° radius) | Blindness | 238.41 | (230.28, 246.54) | 256.22 | (249.02, 263.43) |
| Mild VAI (0.30 < VA [logMAR] ≤ 0.52) | Normal VF | Mild VI | 195.96 | (181.08, 210.85) | 228.48 | (219.29, 237.67) |
| Mild VAI (0.30 < VA [logMAR] ≤ 0.52) | Moderate VFI (20° radius) | Severe VI | 177.78 | (162.26, 193.30) | 211.19 | (201.39, 220.98) |
| Mild VAI (0.30 < VA [logMAR] ≤ 0.52) | Severe VFI (10° radius) | Blindness | 137.78 | (120.09, 155.46) | 191.15 | (180.58, 201.71) |
| Moderate VAI (VA [logMAR] > 0.52) | Normal VF | Moderate VI | — | — | 218.15 | (211.23, 225.07) |
| Moderate VAI (VA [logMAR] > 0.52) | Moderate VFI (20° radius) | Severe VI | — | — | 186.52 | (180.97, 192.06) |
| Moderate VAI (VA [logMAR] > 0.52) | Severe VFI (10° radius) | Blindness | — | — | 152.96 | (141.06, 164.87) |

cpm = characters per minute; CI = confidence interval; MRS = maximum reading speed; RS = reading speed; VA = visual acuity; VAI = visual acuity impairment; VF = visual field; VFI = visual field impairment; VI = visual impairment

RS/MRS are expressed as cpm. VA is binocular and expressed in logMAR. VF restriction angles denote the radius of the central field (20° and 10°). WHO visual-impairment labels were assigned for descriptive purposes using the more severe criterion indicated by VA or VF. RS in the Moderate VAI level was not reliably measurable at 40 cm across VF conditions and was therefore not included in RS analyses.

**Supplementary Figure 1** Representative schematic of the Chinese Reading Acuity Chart. The chart comprises sentences printed in progressively smaller character sizes. Each sentence contains 30 Chinese characters, with adjacent print sizes differing by 0.1 log units. Examples shown illustrate the variation in font size and the corresponding visual-acuity scaling information provided on the chart.


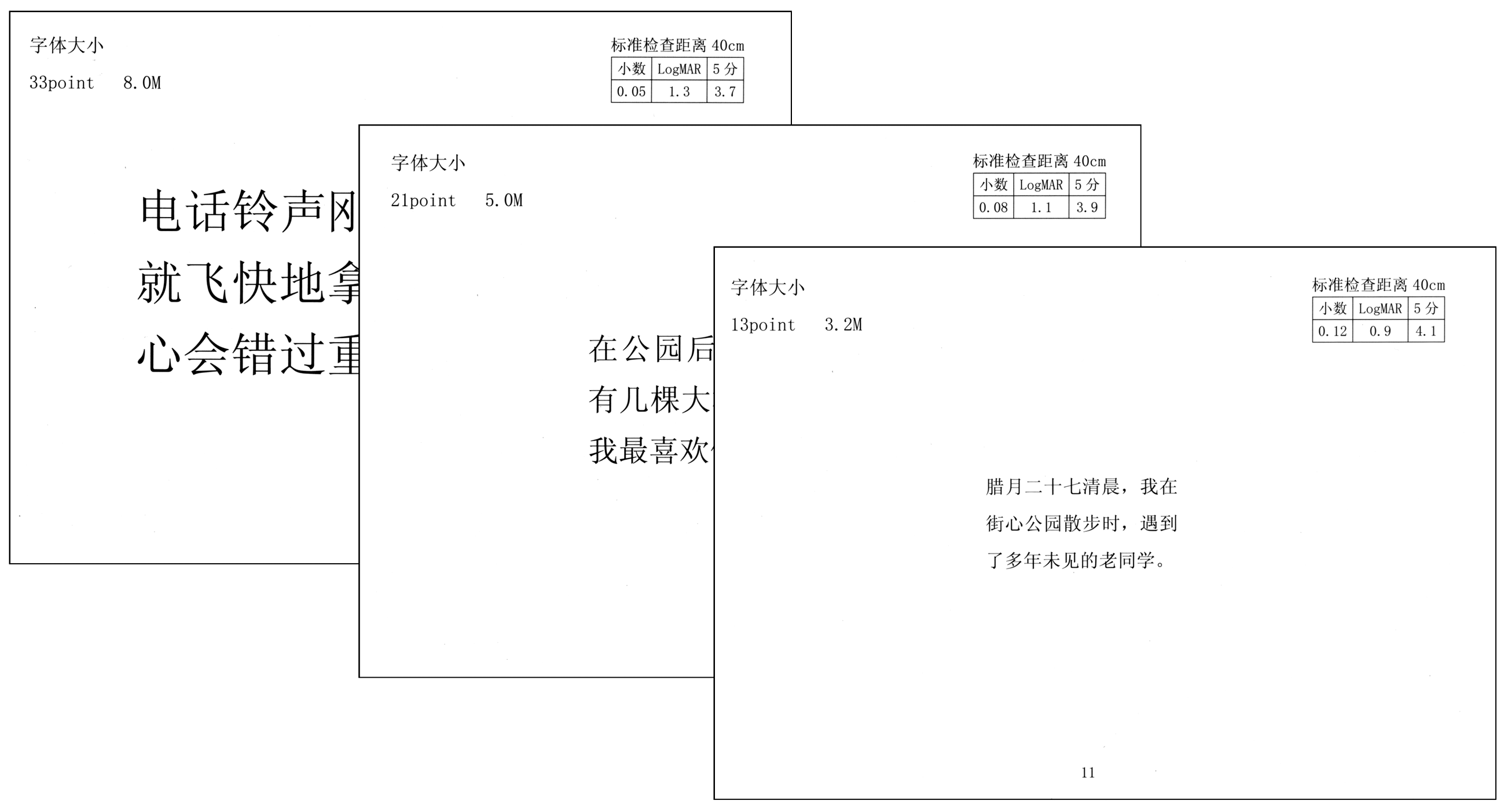

Supplement: Supplementary file 1 — Supplementary file1 [file 40662_2026_497_MOESM1_ESM.docx]
